# Large macular hole and autologous retinal transplantation: a systematic review and meta-analysis

**DOI:** 10.1186/s40942-024-00573-1

**Published:** 2024-08-22

**Authors:** Mário Hanai, Dillan Cunha Amaral, Raiza Jacometti, Eduardo Henrique Cassins Aguiar, Fernando Cotrim Gomes, Laura Goldfarb Cyrino, Milton Ruiz Alves, Mário Luiz Ribeiro Monteiro, Raphaela Masetto Fuganti, Antonio Marcelo Barbante Casella, Ricardo Noguera Louzada

**Affiliations:** 1 Faculty of Medicine, Federal University of Rio de Janeiro, Rio de Janeiro, RJ Brazil; 2 Division of Ophthalmology and the Laboratory for Investigation in Ophthalmology (LIM-33), Faculty of Medicine, University of São Paulo, São Paulo, SP Brazil; 3 Faculty of Medicine, Federal University of Minas Gerais, Belo Horizonte, MG Brazil; 4 Faculty of Medicine, State University of Londrina, Londrina, PR Brazil; 5 Instituto de Olhos São Sebastião, Largo do Machado 54, 1208, Rio de Janeiro, RJ 22221-020 Brazil

**Keywords:** Macular hole, Vitrectomy, Autologous neurosensory retinal transplantation, Neurosensory retinal graft, Autologous retinal transplantation, Large macular hole, Recurrent macular hole, Persistent macular hole, Refractory macular hole, Retinal transplantation

## Abstract

**Introduction:**

Macular holes are breaks in the retinal tissue at the center of the macula, affecting central vision. The standard treatment involves vitrectomy with membrane peeling and gas tamponade. However, for larger or chronic holes, alternative techniques like autologous retinal graft have emerged. This meta-analysis evaluates the efficacy and safety of retinal transplantation in managing large macular holes.

**Methods:**

We conducted a systematic review and meta-analysis following PRISMA guidelines. The study was prospectively registered in PROSPERO (CRD42024504801). We searched PubMed, Web of Science, Cochrane, and Embase databases for observational studies including individuals with large macular holes with or without retinal detachments and retinal transplantation as the main therapy. We used a random-effects model to compute the mean difference with 95% confidence intervals and performed statistical analysis using R software.

**Results:**

We conducted a comprehensive analysis of 19 studies involving 322 patients diagnosed with various types of macular holes (MHs). These included cohorts with refractory MH, high myopia associated with MH, primary MH, and MH with retinal detachment (RD). The findings were promising, revealing an overall closure rate of 94% of cases (95% CI 88–98, I^2^ = 20%). Moreover, there was a significant improvement in postoperative visual acuity across all subgroups, averaging 0.45 (95% CI 0.33–0.58 ; I^2^ = 72%; *p* < 0.01) overall. However, complications occurred with an overall incidence rate of 15% (95% CI 7–25; I^2^ = 59%).

**Conclusion:**

ART for large MH shows promising results, including significant improvements in visual acuity and a high rate of MH closure with low complication risks overall and for subgroups.

## Introduction

A macular hole (MH) is a complete break in the retinal tissue located at the central part of the macula, and it affects the central vision and causes metamorphopsia [1–4]. MH can be associated with diabetic retinopathy, pathologic myopia, and other eye conditions, but most MH cases develop without a clear secondary cause, known as idiopathic MH as idiopathic macular hole (IMH) [2, 5, 6]. Approximately 7.8 per 100.000 persons yearly are newly diagnosed, and the most critical risk factors associated with MH idiopathic formation are older age and female sex; approximately two-thirds of patients are females [5, 7]. There are several hypotheses on the development of IMH, and the most accepted is related to Vitreous Macular Traction [5, 7]. This entity has been classified clinically since 1994 by Gass and, more recently, based on OCT imaging by the International Vitreomacular Traction Study (IVTS) Group [8, 9]. According to the IVTS group, large macular holes are characterized by a narrowest horizontal dimension exceeding 400 micrometers [10]. Persistent MHs are those that fail to close after primary surgery, and recurrent MHs are those that reopen after successful closure [11].

The primary challenges in managing this condition include its significant size, concurrent retinal detachment (RD), duration of the MH, and likelihood of recurrence. Vitrectomy, along with internal limiting membrane (ILM) peeling and gas tamponade, has become the established standard of care for treating full-thickness MHs. [3, 12]. This method effectively closes over 90% of MH [12]. However, it can be insufficient for large and chronic macular holes. The recurrence rate ranges from 4.8 to 9.2%, typically occurring between 12 and 15 months post-operatively [13]. Therefore, nowadays, the literature focuses on an alternative technique to manage these challenging cases [11]. Several modified surgical approaches have been documented for addressing it, such as utilizing a free internal limiting membrane (ILM) flap, employing an amniotic membrane graft (AMG), using the anterior lens capsule, and lastly, the recent technique of implementing an autologous retinal graft (ARG) [14–16]. The ARG, or autologous retinal transplantation (ART), was proposed in 2016 by Grewal D. and Mahmoud T [17]. Since then, different centers worldwide have been performing this new modality and, most of all, shown promising results with good anatomical and functional outcomes [18–33]. Most studies demonstrated that ART offers a high rate of anatomic success and, at the same time, is considered safe, with few intra and postoperative complications, showing benefits over the another treatment methods in large and complex MH [26, 34, 35]. Nevertheless, the advantages of this technique, in comparison to others, as well as the surgery-related results and complications, are still controversial.

Therefore, consolidating the results of this alternative method for challenging cases of large MH is essential to ascertain the device that could offer superior effectiveness and safety. To address this significant knowledge gap, we conducted a comprehensive systematic review and meta-analysis to evaluate the efficacy and safety of ART for large macular holes.

## Materials and methods

### Protocol and search strategy

We systematically searched the PubMed, Cochrane Library, Embase, and Web of Science databases. This study was registered in the International Prospective Register of Systematic Reviews (PROSPERO; CRD42024531731). Our search strategy was carefully crafted to thoroughly investigate the topic utilizing a comprehensive combination of relevant keywords. The specific keywords employed in our search included: (“macular hole” OR “macular holes”) AND (“Autologous retinal transplantations” OR “autologous neurosensory transplants” OR “transplant” OR “transplantation” OR “retinal transplant” OR “retinal transplants” OR “retinal graft” OR “retinal grafts” OR “free flap transplantations”). We started our search on 6 March 2024 and completed it on 20 March 2024, identifying studies published from 1972 to 2024. This meticulous approach ensured we obtained the most pertinent and reliable information, empowering us to present a well-founded and in-depth analysis of the subject matter.

### Eligibility criteria

The inclusion criteria of this study were as follows: (1) Participants: individuals (> 18 years) with large MH with or without RD (2) Intervention: ART; (3) Type of study: non-randomized studies and case series (4) Any refractory MH. The exclusion criteria were as follows: (1) animal studies; (2) case reports, abstracts, editorials, letters, and conference proceedings without efficient data; and (3) studies that previously selected patients with ART-specific complications; (4) studies that did not report individual results on ART surgeries. This exclusion was implemented to ensure that only high-quality studies were included in the analysis.

### Outcomes

Our study aimed to evaluate multiple endpoints, encompassing the endpoints of (1) Best-visual acuity (LogMAR) change; (2) MH closure; and (3) Complications rates. The complications included in included studies and subjected to our statistical analysis were endophthalmitis, high intraocular pressure necessitating clinical intervention, presence of subretinal perfluorocarbon, high myopia, choroidal neovascularization, graft dislocation, intraoperative bleeding, incorrect graft transplantation, macular edema, retinal detachment, reactive pigment epithelial damage, reactive pigment epithelial hyperplasia, total complications, uveitis, vitreous hemorrhage, and vitreoretinopathy.

### Study selection

We imported search results into the Zotero software, and duplicated records were excluded. Two independent authors (M.H. and E.A.) applied eligibility criteria to screen the titles and abstracts. After that, the full text of potentially eligible studies was appraised. Any disagreements were resolved by contacting the senior author (R.L.).

### Data extraction

Two authors (M.H. and E.A.) extracted the following data from selected studies: country, study design, number of patients and eyes allocated for each arm, time to follow-up, and the main patient’s baseline characteristics. The same authors also collected pre-specified baseline characteristics and outcome data and recorded them in an Excel template.

### Quality assessment

To detect biases and assess the quality of the studies, two independent reviewers (M.H. and E.A.) used the Joanna Briggs Institute (JBI) checklist for critical appraisal [36]. This tool has ten items, each with four response options: Yes, No, Unclear, or Not applicable. We allocated one point for each item, so the overall score for each study ranged from zero to ten. In this review, studies were categorized into three quality levels: low (zero to four points), medium (five to seven points), and high (eight to ten points). No eligible study was excluded due to quality. Disagreements were resolved by consensus after discussing the reasons for the discrepancies.

### Statistical analysis

This systematic review and meta-analysis were performed per the Cochrane Collaboration and the Preferred Reporting Items for Systematic Reviews and Meta-Analysis (PRISMA) [37] statement guideline. We conducted a proportional meta-analysis pooling the data with the function “metaprop” and “metacont”, included in the packages “meta”, “metafor” in R for efficacy and safety outcomes [38]. The calculation of combined means and standard deviations was performed using Cochrane’s formula and adhering to recommended guidelines [39, 40]. Categorical endpoints were assessed using Relative Risk (RR) with corresponding 95% confidence intervals (CIs), while continuous outcomes were evaluated using Mean Difference (MD). I^2^ statistics were used to assess heterogeneity; I^2^ > 50% was considered substantial heterogeneity. We used DerSimonian and Laird random effects models for all endpoints [41]. The *p* < 0.05 was considered statistically significant. When many proportions are equal to zero or one, we transform our data using double-arcsine transformation (Freeman-Tukey double-arscine) [42]. Statistical analysis was performed using the software R (version 4.2.3, R Foundation for Statistical Computing, Vienna, Austria) [43].

### Publication bias assessment

We evaluated publication bias among the included studies using Egger’s test and Funnel plot analysis, where a significance level of *P* < 0.05 indicated a notable difference [44, 45].

## Results

**Fig. 1:**
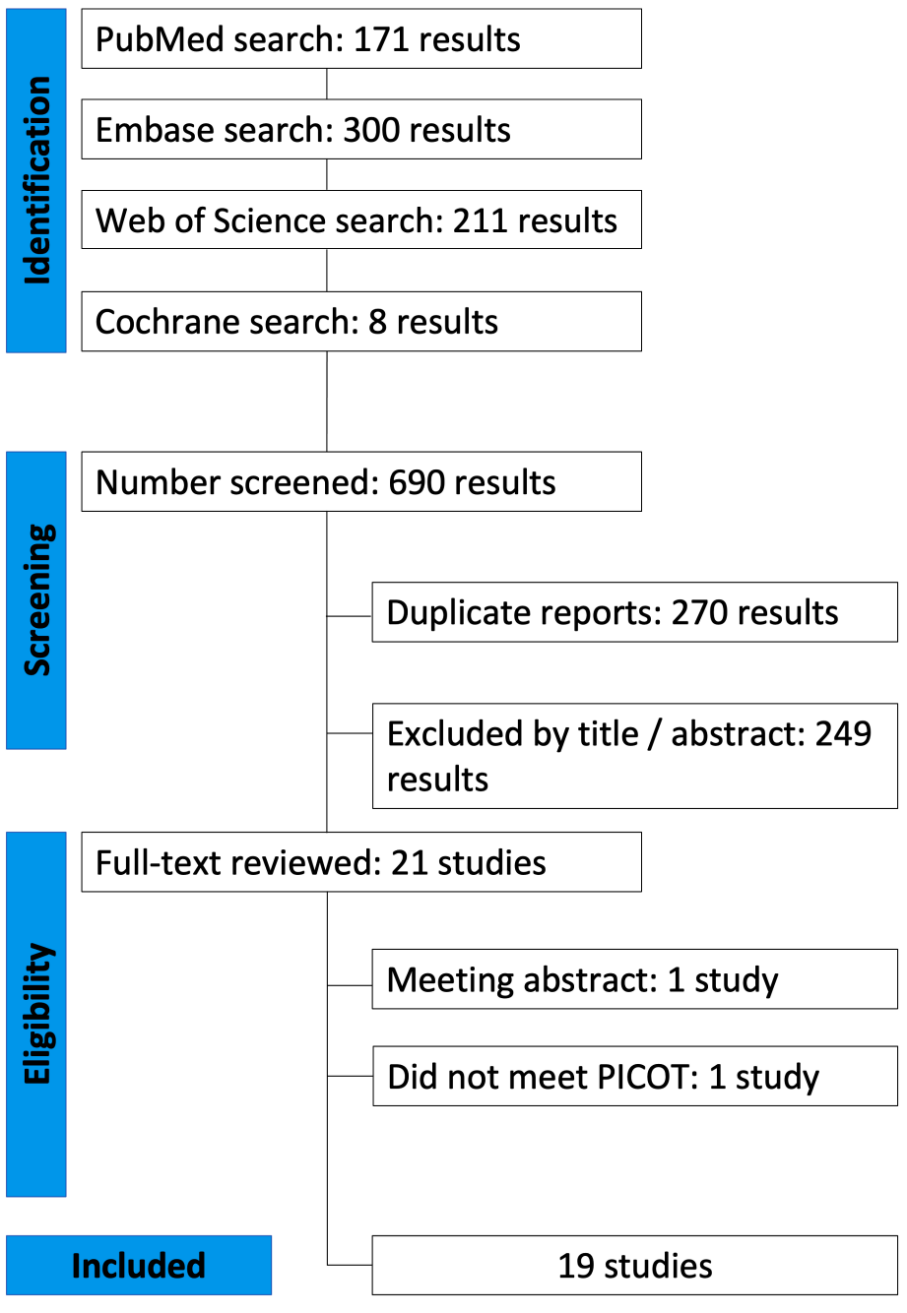
PRISMA flow diagram of study screening and selection process

The search strategy yielded a total of 690 abstracts and/or manuscripts. After excluding 270 duplicated studies and 401 studies that weren’t related to the research question, 19 studies were included [18–32, 46–49] (Fig. 1). Sixteen studies included were retrospective and three were prospective, the studies reported groups with refractory MH (208), high myopia associated with MH (17), primary MH (71), and MH with RD (26) (Table 1). A non-overlapping population of 322 patients was included in this study. Among the studies that reported the female-to-male ratio, 42% of the patients were male, while two studies did not provide this information. Other baseline characteristics are in Table 1.

**Table 1:** Baseline characteristics

Author	Year	Country District	Design	Disease Status	Sample Size	Female/Male ratio	Mean Age, years	Mean Follow-up Time, months	Refractory consideration	Type of surgery	Lens Status Before	Mean axial length (mm)	Mean maximum basal diameter macular hole, µm (Range)	Mean minimal inner-opening diameter macular Hole µm (range)	Size Of the graft compare to the size of The MH	Tamponade agent	Time to remove Silicon Oil, months	Time in Prone Position, days
Wu et al.	2018	Taiwan	R	Refractory	6	4/2	59.0 ± 9.9	25.2	Previous PPV + ILM removal	PPV + LP to harvest the graft + ART under ABC + AFX + SF6 gas	Pseudophakic	25.63	978.5 ± 441	538 ± 184	1x	NA	NA	7
Chang et al.	2019	Taiwan	R	Refractory	10	7/3	64.9 ± 11.5	12	≥ 2 ILM peeling surgeries	PPV + LP to harvest the graft + ART under autologous blood clot + AFX + SO	NA	NA	1404.2 ± 562.9	NA	1,5-2x	SO	6	14
Ding et al.	2019	China	R	Refractory	5	2/3	35.4 ± 18.72	6	Vitrectomy with ILM removal or transplantation	PPV + LP + ART under heavy water (deuterium oxide) + Retinopexy + AFX + SO	Phakic 2 / Pseudophakic 3	29.26	NA	NA	NA	SO	NA	NA
Grewal et al.	2019	USA/Italy/ Japan	R	Refractory	41	27/14	61 ± 14.9	11.1	≥ 1 prior surgery	PPV + removal residual peripheral vitreous + ART under PFO + C3F8 gas or SO or short-term PFO	Phakic 11 / Pseudophakic 30	27.85	1468.1 ± 656.4	825 ± 422.5	2x	SO, C3F8 gas or short-term PFO	1–3	7 (when PFC used as tamponade pacients were positioned supine)
Tanaka et al.	2019	Japan	R	Primary	7	2/5	71.42 ± 11.5	16	Not applicable	Vitrectomy + ART + PFO with diamond-dust scraper or Flexloop + residual ILM removal + Retinopexy + AFX with SF6	NA	24.56	1214 ± 219.5	661 ± 144.5	1,500 μm	SF6	NA	3
Yamada et al.	2020	Japan	R	Refractory/ Refractory with High Myopia	4	4/0	75.8 ± 7.7	≥ 6	≥ 1 prior surgery such as ILM peeling and ILM transplantation	Vitrectomy + ART + AFX + SO (1000 centistokes)	Pseudophakic	27.85	1420 ± 338	628 ± 348	2x	SO (1000 cs)	NA	2
Li et al.	2020	China	R	Refractory with High Myopia	10	8/2	50.3 ± 11.50	≥ 3	Previous ILM peeling	PPV + residual ILM removal + ART under PFO + SO	Aphakic 8 / Pseudophakic 2	31.42	1192.60 ± 467.51	NA	NA	SO	3–6	30
Rojas-Juarez et al.	2020	Mexico	P	Refractory	13	6/7	67.15 ± 12.28	12	≥ 1 prior surgery	PPV + residual ILM removal + ART under PFO + Retinopexy + AFX + SO (5000 centistokes)	NA	25.01	1615.38 ± 689.19	964.08 ± 709.77	1.2x to 1.5x	SO (5000cs)	3–6	NA
Moysidis et al.	2021	Worldwide	R	Primary/Refractory/Regmathogenous	130	75/55	63 ± 6.3	8.6	ILM peeling previously	PPV + ART under PFO + short-term PFO or AFX then SO or SF6 gas	In (P): Phakic (77%) / Pseudophakic (23%); In (R) 43% Phakic / 57% Pseudophakic	24.8	1470 ± 165	837 ± 94	NA	PFO (20%) or SO(60%) or diluted gas (SF6 or PFC) (20%)	NA	7 if gas or silicon oil (supine if PFO)
Sonmez et al.	2021	Turkey	R	Refractory	7	4/3	60.6 ± 8.6	18.8	≥ 2 unsuccessful surgeries (extended ILM peeling, free ILM flap transplantation or retina expansion technique)	PPV + ART under PFO + PFO-SO (1000 centistokes) exchange	Pseudophakic	NA	1146.7 ± 413.7	788.9 ± 148.8	2-3x	SO (1000 cs)	3–6	7
Dhami et al.	2022	India	R	Refractory or Retinal Detachment	22	11/11	54.59 ± 13.68	6–18	Vitrectomy + ILM peeling previously	3 methods: 1º PPV + phacoemulsification with iol implantation (if significant cataract) + epiretinal fibrous tissue residual dissect + ART under PFO + AFX + SO or non-expansile gas. 2º membrane loop was used to lift the retinal flap + endodiathermy barrage + the rest is the same. 3º specially design retinal punch was used to create free ARG + no endodiathermy used + the rest is the same.	Phakic 7 / Pseudophakic 14 / Aphakic 1	NA	NA	1103.67 ± 310.09	NA	SO in 7 / gas in 15	NA	7
Takeuchi et al.	2021	Japan	P	Refractory with High Myopia	5	4/1	68.8 ± 15	13.6	Multiples surgeries previously	PPV + ART under PFO + SO	Phakic 1 / Pseudophakic 4	29.7	1504 ± 684	1175 ± 340	1.5-2x	SO	NA	14
Kitahaka et al.	2022	Japan	R	Primary	17	8/9	63.2 ± 18.8	13.6	Not applicable	Vitrecomy + ART under PFO + Retinopexy + AFX with SF6	Phakis 14 / Pseudophakic 3	23.62	1295.8 ± 276.9	632 ± 120.9	NA	SF6	NA	NA
Lee et al.	2022	Taiwan	R	Refractory	9	6/3	63.6 ± 11.4	24	≥ 2 unsuccessful surgeries (ILM peeling, extended ILM peeling or autologous free ILM flap transplantation)	PPV + ART with ABC or small amount of Viscoat + AFX + SO	NA	NA	1437.6 ± 719.5	NA	1,5-2x	SO	6	NA
Lorenzi et al.	2022	France/Italy	R	Refractory	10	NA	68 ± 11	12	ILM peeling previously	PPV + subretinal fluid injection to harvest the graft + ART	Pseudophakic	26.30	975 ± 421	630 ± 286	2x	SO 7 / gas 3	NA	5
Lumi et al.	2022	Slovenia	P	Refractory/ Large MH with Retinal Detachment	4	2/2	71.75 ± 10.5	17	Multiple vitrectomies and ILM peeling	PPV + ART subretinal or epiretinal + SO (2000 cs)	Pseudophakic	23.87	1801 ± 861.5	827.75 ± 271.5	1,5 − 1,8x	SO (2000 cs)	3–8	NA
Morales-Canton et al.	2023	Mexico	R	Refractory	6	3/3	63.7 ± 14.3	24	≥ 1 prior PPV surgery	PPV + ART under PFO + AFX	NA	NA	2443 ± 2956	909 ± 635	2x	NA	NA	NA
Zgolli et al.	2023	Tunisia	R	Primary	11	5/6	56.6 ± 10.33	3	Not applicable	Central and peripheral vitrectomy + ART under PFO + Retinopexy + air-PFO exchange + SO (Polydimethylsiloxane, Baush and Lomb Oxane 1300)	Phakic 9 / Pseudophakic 2	NA	NA	NA	NA	SO (Polydimethylsiloxane, Baush and Lomb Oxane^®^ 1300)	NA	NA
Park et al.	2024	Worldwide	R	Primary and Refractory of MTMH	5	NA	68.1	10	Unsuccessful ILM peels and one with unsuccessful previous ART	Vitrectomy + ART + SF6 gas or PFO + SF6-PFO exchange	Phakic 1 / Pseudophakic 4	NA	848.6 ± 348.5	390.2 ± 203.7	NA	SF6 gas 20% or PFO 80%	NA	NA

ABC: autologous blood clot; AFX: air-fluid exchange; ARG: autologous retinal graft; ART: autologous retinal transplantation; ILM: internal limiting membrane; IOL: intraocular lens; LP: laser photocoagulation; MH: macular hole; MHRD: macular hole retinal detachment; MTMH macular telangiectasia type 2 associated full-thickness macular hole; NA: not available; P: prospective; PFO: perfluor-n-octane; PPV: pars plana vitrectomy; R: retrospective; SF6: sulfur-hexachloride 6; SO: silicon oil

**Fig. 2:**
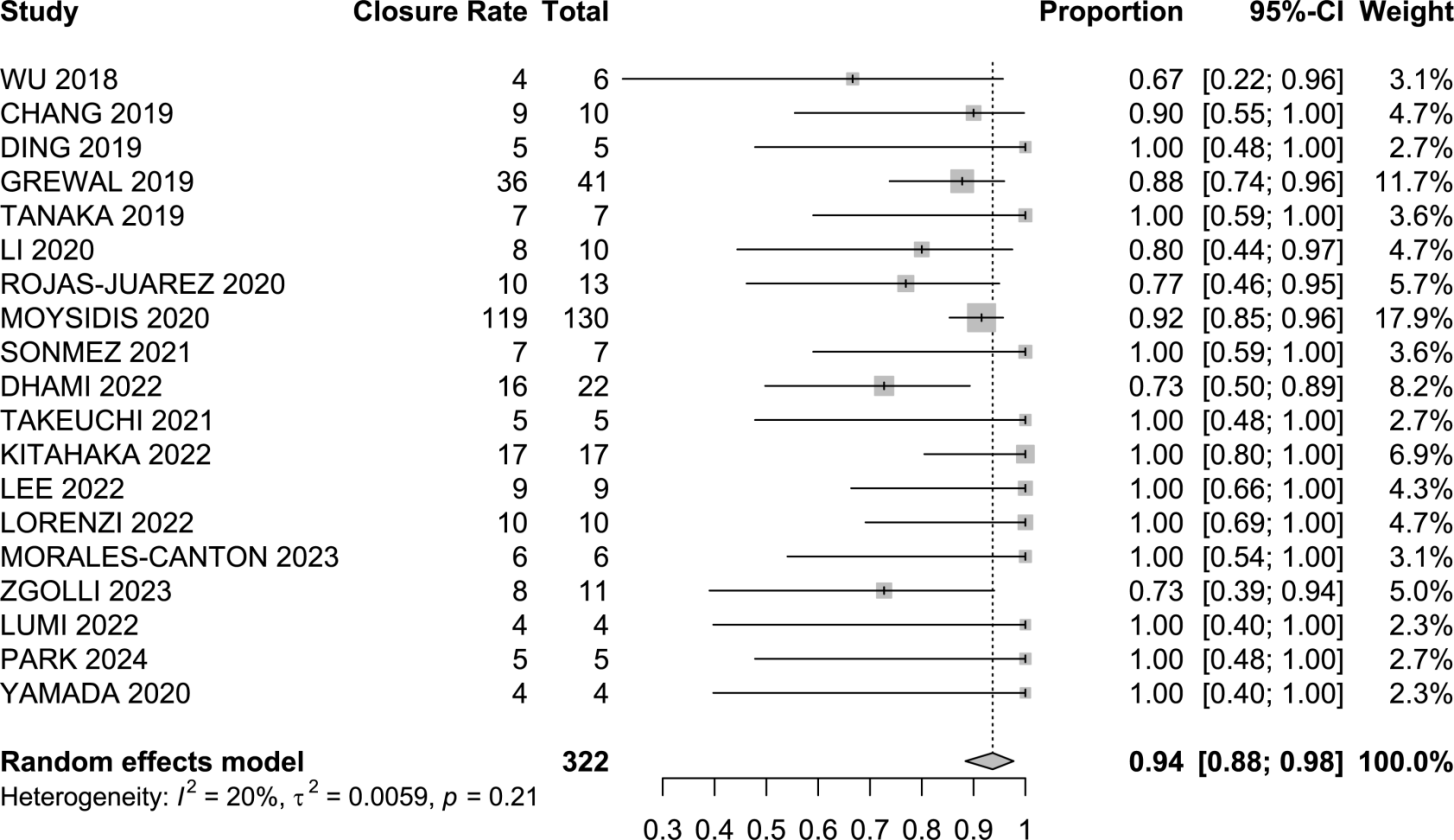
Macular hole closure forest plot

The primary outcome of closure rate was observed in 94% of cases (95% CI 88–98, I^2^ = 20%; Fig. 2). In the refractory macular hole subanalysis, it was observed in 93% of cases (95% CI 85–99, I^2^ = 5%), while for primary macular hole, it was reported in 91% of cases (95% CI 79–99, I^2^ = 11%). For the refractory group presenting with hyperopic patients, it was present in 98% of cases as well (95% CI 87–100, I^2^ = 9%), and for those presenting with retinal detachment, it was present in 88% (95% CI 60–100, I^2^ = 76%) (Fig. 2).

**Fig. 3:**
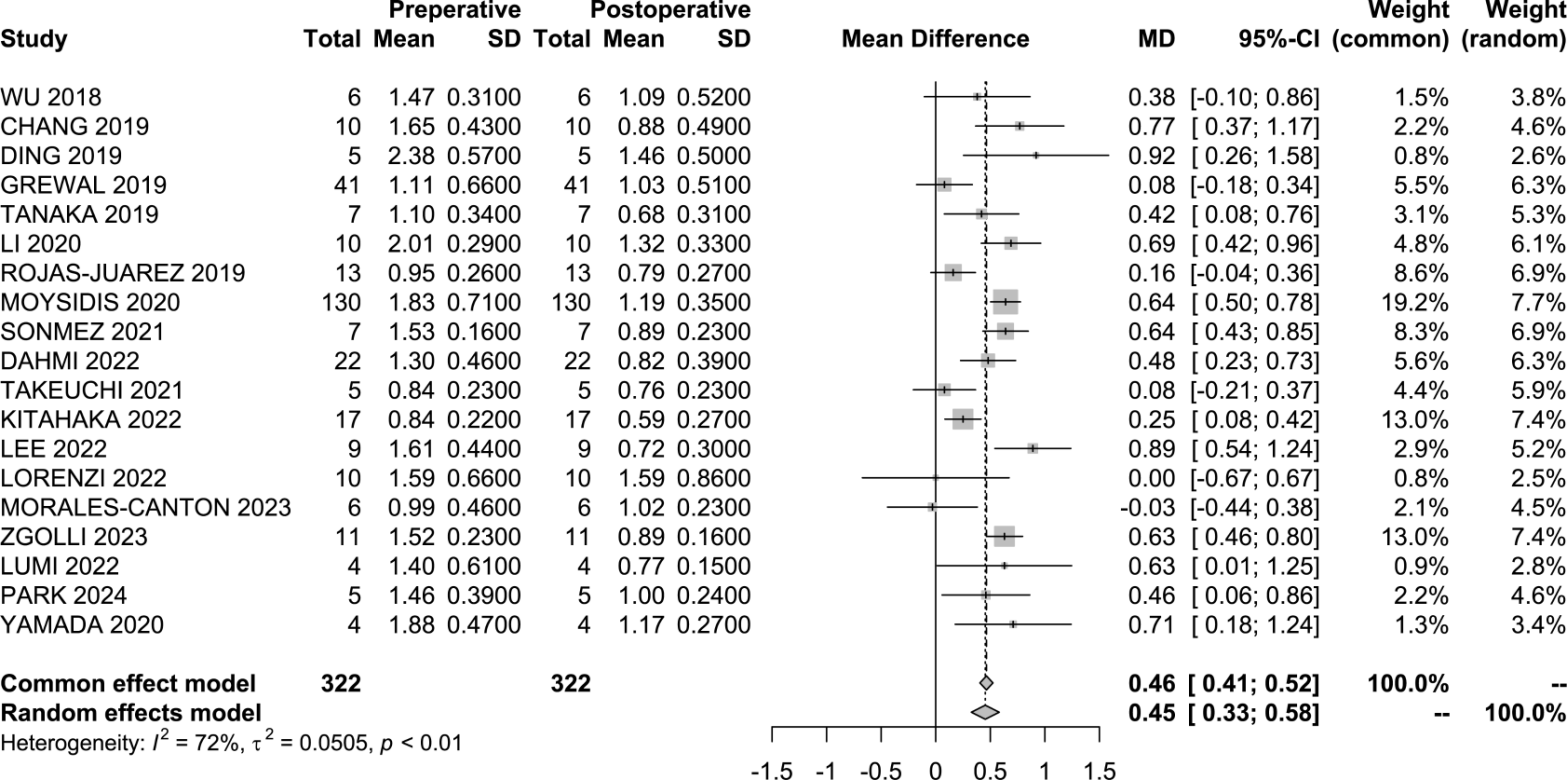
Visual acuity changes forest plot

Postoperative visual acuity (VA) was statistically significantly higher in every subgroup analysis. For the overall cohort, the mean difference in logMAR between pre-operative and post-operative observations was 0.45 (95% CI 0.33–0.58 ; I^2^ = 72%; *p* < 0.01; Fig. 3). The primary macular hole group changed by 0.57 (95%CI 0.15–0.98; I^2^ = 92%; *p* < 0.01). For the refractory macular hole it changed by 0.38 (95%CI 0.25–0.51; I^2^ = 59%; *p* < 0.01), for those with refractory macular assessed as highly myopic it varied by 0.19 (95% CI 0.07–0.31; I^2^ = 17%, *p* < 0.0001), and for those with macular holes and retinal detachment, it decreased by 0.78 (95% CI 0.49–1.06; I^2^ = 86%; *p* < 0.01).

**Fig. 4:**
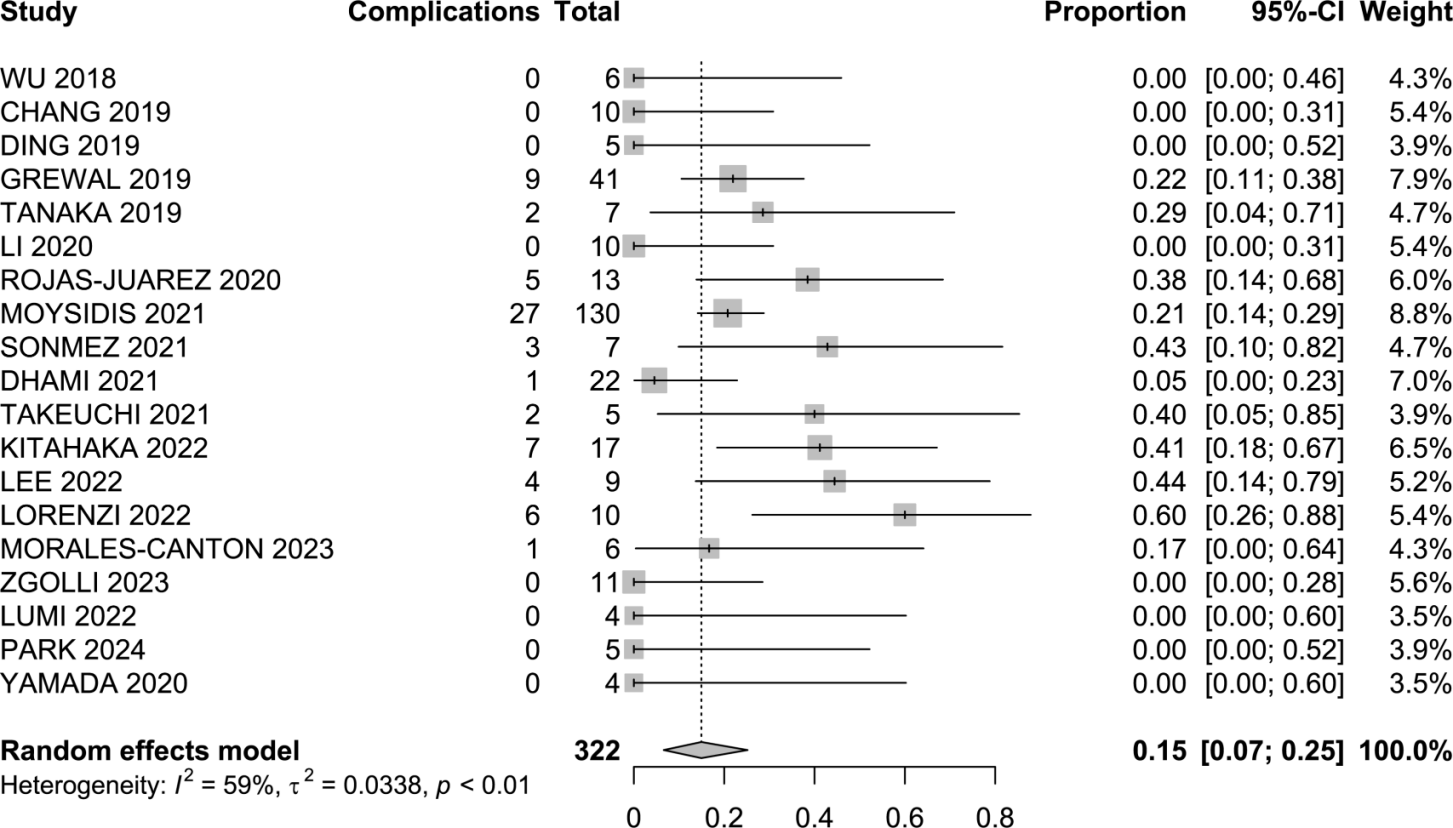
Complications forest plot

The total incidence of complications was 15% (95% CI 7–25; I^2^ = 59%; Fig. 4). In the primary macular hole group, it amounted to 23% (95% CI 3–51; I^2^ = 73%). The reported rate for those presenting with refractory macular holes was 16% (95% CI 7–28; I^2^ = 50%). For the refractory macular hole in the highly myopic patients’ group, there was a prevalence of 4% (95% CI 0–20; I^2^ = 36%), and for those presenting with associated retinal detachment, it was 16% (95% CI 0–71; I^2^ = 89%). Table 2 reported the detailed incidence of all complications included in each study.

**Table 2:** Postoperative complications for all studies

Study_year	sub	*n*	RD	h-IOP	E	S-PFC	CNV	VH	IB	IGT	ME	VP	RPEH	RPED	UV	GD	TC
WU 2018	Refractory	6	0	0	0	0	0	0	0	0	0	0	0	0	0	0	0
CHANG 2019	Refractory	10	0	0	0	0	0	0	0	0	0	0	0	0	0	0	0
DING 2019	Refractory	1	0	0	0	0	0	0	0	0	0	0	0	0	0	0	0
DING 2019	Refractory+ HM	4	0	0	0	0	0	0	0	0	0	0	0	0	0	0	0
GREWAL 2019	Refractory	41	1	0	0	0	0	1	0	0	7	0	0	0	0	0	9
TANAKA 2019	Primary	7	0	0	0	0	0	0	0	1	1	0	0	0	0	0	2
LI 2020	Refractory+HM	10	0	*	0	0	0	0	0	0	0	0	0	0	0	0	0
ROJAS-JUAREZ 2020	Refractory	13	0	3	0	0	0	0	0	0	0	1	1	0	0	0	5
MOYSIDIS 2021	Primary	35	0	1	0	0	0	0	0	0	0	1	0	0	0	1	3
MOYSIDIS 2021	MH + RD	19	4	1	0	2	0	0	0	0	0	0	0	0	0	1	8
MOYSIDIS 2021	Refractory	76	1	2	1	0	0	0	5	0	0	0	0	2	0	3	14
SONMEZ 2021	Refractory	7	1	0	0	0	0	0	0	0	1	0	1	0	0	0	3
DHAMI 2021	Refractory+HM	22	0	0	0	0	0	0	0	0	0	0	0	0	0	1	1
TAKEUCHI 2021	Refractory+ HM	5	0	0	0	0	2	0	0	0	0	0	0	0	0	0	2
KITAHAKA 2022	Primary	17	0	0	0	0	0	0	0	0	7	0	0	0	0	0	7
LEE 2022	Refractory	9	0	0	0	0	0	0	0	0	4	0	0	0	0	0	4
LORENZI 2022	Refractory	10	0	0	0	0	0	0	0	0	5	0	0	0	1	0	6**
MORALES-CANTON 2023	Refractory	6	0	0	0	0	0	0	0	0	0	0	0	0	1	0	1
ZGOLLI 2023	MH+ RD	11	0	0	0	0	0	0	0	0	0	0	0	0	0	0	0
LUMI 2022	Refractory	4	0	0	0	0	0	0	0	0	0	0	0	0	0	0	0
PARK 2024	Refractory	5	0	0	0	0	0	0	0	0	0	0	0	0	0	0	0
YAMADA 2020	Refractory	4	0	0	0	0	0	0	0	0	0	0	0	0	0	0	0
TOTAL		322	7	4	1	2	2	1	5	1	6	2	1	2	1	6	41

Abbreviations SUB, subgroup; E, endophtalmitis; H-IOP, High IOP requiring clinical treatment; S-PFC, subretinal perfluorcarbon HM, high myopia; CNV, choroidal neovascularization; GD, graft dislocation; HM, high myopia; IB, intraoperative bleeding; IGT, incorrectly graft transplantation; ME, macular edema; RD, retinal detachment; RPED, reactive pigment epithelial damage; RPEH, reactive pigment epithelial hyperplasia; TC, total complications; UV, uveitis; VH, vitreous hemorrhage; VP, vitreoretinopathy

* The authors do not provide the exact result

**Excluded postoperative complications preventing fundus imaging and/or requiring further vitreoretinal surgery

**Fig. 5:**
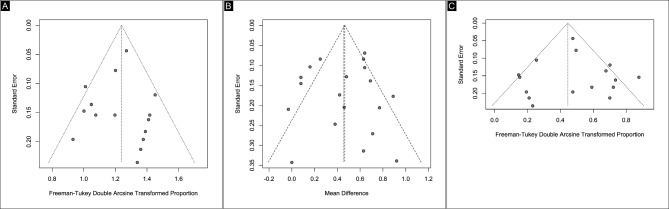
Funnel plots of endpoints. (**A**) Macular hole closure. (**B**) Visual Acuity. (**C**) Complications

**Fig. 6:**
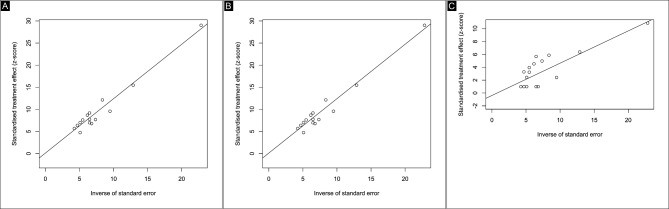
Egger test of endpoints. (**A**) Macular hole closure. (**B**) Visual Acuity. (**C**) Complications

**Fig. 7:**
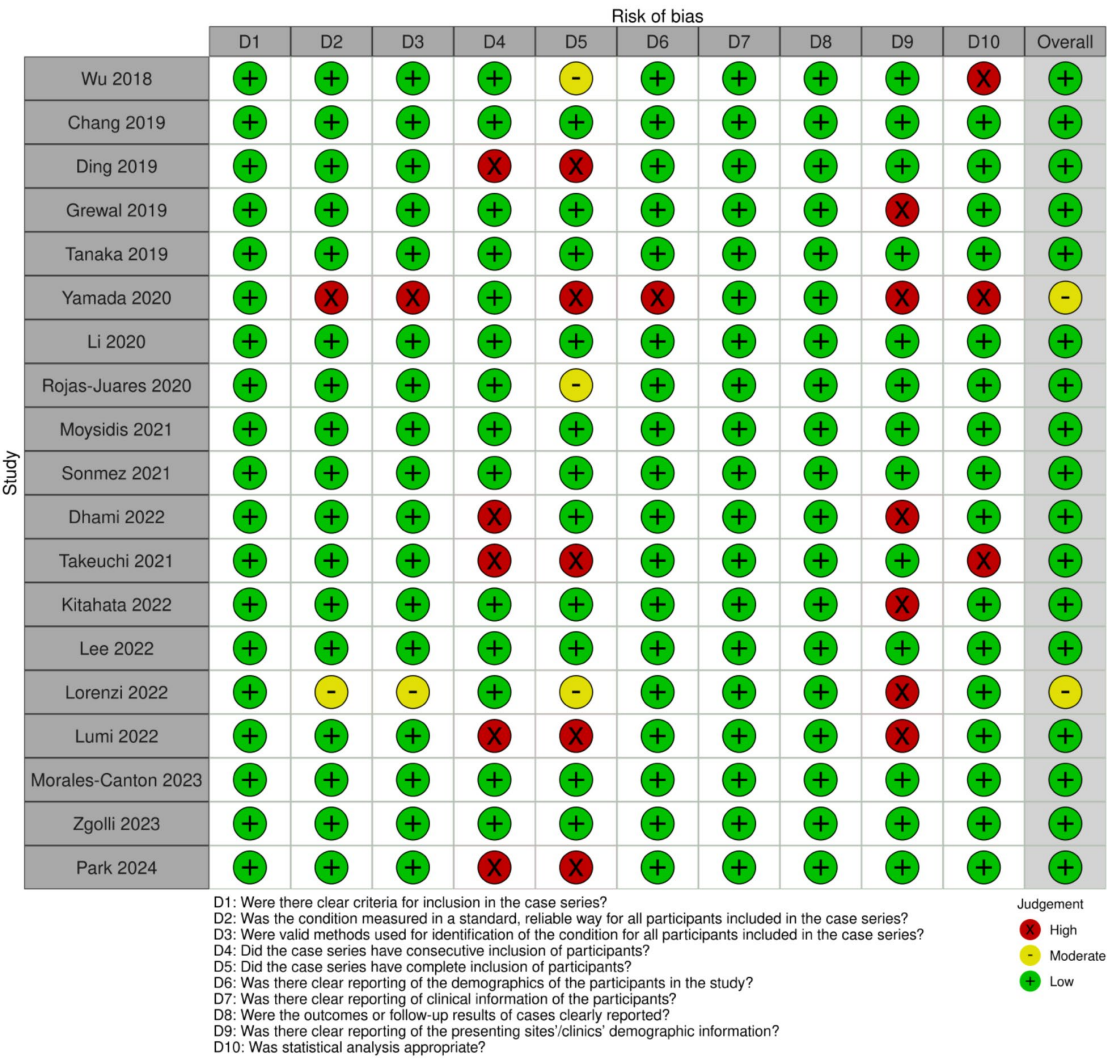
Risk of bias – JBI Critical Appraisal Checklist for Quasi-Experimental Studies

Figures 5 and 6 displayed funnel plots and linear regression tests for funnel plot asymmetry using Egger’s test, respectively. Egger’s test for funnel plot asymmetry found no evidence of potential publication bias in the analysis on MH closure (*p* = 0.8238664), visual acuity (*p* = 0.82392), and complications (*p* = 0.6036). The assessment of the included studies showed a range from low to moderate risk of bias on the JBI checklist. Only two studies were classified as medium quality: Yamada et al. and Lorenzi et al. The Yamada study had several issues contributing to its moderate risk of bias, including inconsistent and unreliable measurement of the condition, inadequately reported methods for identifying the condition, failure to include all eligible participants, lack of clear reporting on demographic information, and inappropriate statistical analysis. The Lorenzi study similarly raised concerns regarding risk and reliability, with uncertainty about consistent and reliable measurement of the condition, unclear reporting of methods for identifying the condition, and inadequate reporting of demographic information. Overall, the other studies were classified as low risk of bias (Fig. 7).

## Discussion

The successful closure rate for cases with larger and longer-standing (more than six months) MH became significantly lower using standard PPV [1]. Surgical options for large and/or refractory MH are limited and several surgical techniques have been reported, such as ILM peeling, inverted flap and free ILM flap transplantation, macular hydrodissection, lens capsular flap transplantation, human amniotic membrane graft, and combined therapies such as ART with RPE and choroid free grafts transplant [50–52]. However, a free ILM flap and capsular flap may not be available for patients with persistent MH even after multiple surgeries. As a result, neurosensory retinal free flap transplantation becomes a reasonable and feasible method for the repair of a refractory MH [10].

To our knowledge, this meta-analysis represents the inaugural examination of surgical interventions for ART in large MH in the literature. We detail the study selection and characteristics, encompassing 19 studies with a total of 322 eyes. The findings reveal a notable success rate in achieving MH closure through surgical ART. Additionally, BCVA improved in general MH cases and subgroup analyses post-surgery. Importantly, the overall rates of complications were lower.

In this meta-analysis, we evaluated an autologous neurosensory retinal transplant and placed it over the MH, resulting in anatomic closure in approximately 94%. The closure rate was observed in 93% of refractory MH and 91% in primary cases. Compared with an ILM flap or a lens capsule flap, autologous neuro-sensory retinal transplantations may have the following advantages. Firstly, the transplanted neurosensory retina is thicker, sturdier tissue than the ILM, and the neurosensory retinal patch does not easily drift away during the gas/liquid exchange process. The patch can be well positioned on the surface of the MH, minimizing potential trauma. Secondly, an autologous neurosensory retina provides a partial retinal structure that will not only act as a scaffold but also serve as a plug to seclude the communication between the vitreous and subretinal space, allowing the subretinal fluid to be gradually excreted by the retinal pigment epithelium pump. Thirdly, the peripheral retina, where it is normally harvested, contains the Müller cells that retain the progenitor properties. These cells have the capacity to migrate to the outer nuclear layer, proliferate, and replace the lost photoreceptor cells [1, 2, 9, 20, 26].

In pathological conditions, studies have shown that Muller’s glia can act as a source of neural progenitor cells, migrating to the outer nuclear layer to replace lost photoreceptors [48]. The flap integrates with the surrounding retina, helping to regenerate the outer layers of the retina and contributing to the successful closure of macular holes, although the ellipsoid zone reconstitution may be incomplete. Ectopic synaptogenesis, which involves the extension of bipolar cell axons to form new connections, is a proposed mechanism for visual improvement [20, 26]. The results of OCT and microperimetry support these functional gains, with notable improvements in color vision and contrast sensitivity after surgery [53].

In addition, the use of autologous neurosensory retinal transplantation has been associated with significant anatomical and functional success rates, with improvements in BCVA and color vision tests, as well as greater contrast sensitivity and microperimetry results [20, 26]. The mean difference in logMAR VA between pre and postoperative observations was 0.45, with a statistically significantly increase in postoperative VA results. A decisive factor affecting postoperative vision is the recovery of the outer retinal structure. In our study, the postoperative VA was statistically significantly higher in every subgroup. An autologous neurosensory retina provides a partial retina structure, and the recovery of the ellipsoid zone and the external limiting membrane have been reported as contributors to VA improvement in MH cases after surgery [5, 20, 26]. Furthermore, Rezende et al. found that among diverse alternative treatments for large MH, only ART significantly improved VA for MH larger than 800 microns [51].

Complication rates were low and varied across studies and different subgroups (Table 2). The overall rate was 15%; however, in many cases involving specific complications, such as the presence of an epiretinal membrane, there was no impact on VA [26]. As surgeons advance in performing and refining the ART technique for MHs, it is essential to be well-versed in the typical ART-specific complications, mitigation strategies, and tips for effectively managing issues such as graft dislocations, perfluoro-n-octane liquid complications, and ART-related retinal detachments. A comprehensive understanding of these challenges aims to reduce subsequent complications and enhance both anatomical and visual outcomes [54]. The ART method has shown promising outcomes with significant efficacy in the pooled analysis. However, it is crucial to acknowledge the technical challenges associated with this treatment approach, such as retinal detachment, graft transportation, the necessity for bimanual techniques, and the use of specialized scissors, which may limit its practical application [17, 35].

Our meta-analysis had limitations. All studies are not randomized, which may result in a risk of selection bias. All studies included did not have a comparison group. As MH has a low occurrence, our analysis included a relatively small number of studies and patients. Even though including 19 studies is a strength, a larger dataset would provide more robust and generalizable conclusions. Different studies may have employed variations in surgical protocols, such as the method of harvesting the graft, the use and timing of silicone oil removal, transferring the graft under PFO or oil to the MH, and using autologous blood cut or viscoelastic. These variations can influence treatment outcomes and introduce heterogeneity. The included studies were conducted in various countries, potentially introducing variability in patient demographics, surgical techniques, and follow-up protocols. This may limit the generalizability of the findings to a broader population.

## Conclusions

In conclusion, this meta-analysis provides a comprehensive assessment into the safety and efficacy of ART surgery for large MH. The study showed that ART surgery emerges as a promising treatment, with a remarkable rate of MH closure and substantial improvements in VA with a lower risk of post-operatory complications. Notably, the outcomes were favorable across various subgroups, including primary, refractory, hyperopic, and those with macular holes associated with retinal detachment, all exhibiting excellent safety and promising results. This highlights the importance of continuing research and refining treatment strategies to enhance the overall quality of care for people affected by MH disease. Further randomized, large and long-term studies with evaluation of factors related to the disease and its surgical treatment and their influence on both anatomical and visual outcomes could provide valuable insights. Rigorous post-operative follow-up, additional surgeries to reposition the flap and further advances in surgical techniques are essential to control these complications and ensure successful results in cases of complex macular holes.

## Data Availability

No datasets were generated or analysed during the current study.
